# Single-step collision-free trajectory planning of biped climbing robots in spatial trusses

**DOI:** 10.1186/s40638-016-0033-3

**Published:** 2016-02-24

**Authors:** Haifei Zhu, Yisheng Guan, Shengjun Chen, Manjia Su, Hong Zhang

**Affiliations:** Biomimetic and Intelligent Robotics Lab (BIRL), School of Electro-mechanical Engineering, Guangdong University of Technology, Hi-education Mega Center, Guangzhou, 510006 China; Department of Computing Science, University of Alberta, Edmonton, AB T6G 2E8 Canada

**Keywords:** Biped climbing robots, Motion planning, Collision avoidance, Path smoothing, Rapidly-exploring random tree

## Abstract

For a biped climbing robot with dual grippers to climb poles, trusses or trees, feasible collision-free climbing motion is inevitable and essential. In this paper, we utilize the sampling-based algorithm, Bi-RRT, to plan single-step collision-free motion for biped climbing robots in spatial trusses. To deal with the orientation limit of a 5-DoF biped climbing robot, a new state representation along with corresponding operations including sampling, metric calculation and interpolation is presented. A simple but effective model of a biped climbing robot in trusses is proposed, through which the motion planning of one climbing cycle is transformed to that of a manipulator. In addition, the pre- and post-processes are introduced to expedite the convergence of the Bi-RRT algorithm and to ensure the safe motion of the climbing robot near poles as well. The piecewise linear paths are smoothed by utilizing cubic B-spline curve fitting. The effectiveness and efficiency of the presented Bi-RRT algorithm for climbing motion planning are verified by simulations.

## Background

To release workers from tedious and dangerous high-rise tasks in truss-type environments, such as inspecting or spray-painting the frame of gymnasiums, airports and large bridges, and so on, robots able to autonomously climb poles are ideal solutions with a lot of benefits. Motivated by this, a variety of pole-climbing robots including UT-PCR [1], CPR [2], Shady3D [3], Climbot [4] and 3DCLIMBER [5] have been developed. Among them, biped pole-climbing robots (BiPCRs), whose main bodies are usually serial arms with multiple degrees of freedom (DoFs) and both ends are mounted with attaching devices, are considered to be outstanding, thanks to their high mobility in terms of multiple climbing gaits, strong ability to transit between poles and to overcome obstacles.

The ultimate goal of developing BiPCRs is to autonomously carry out high-rise tasks in place of humans. To this end, autonomous climbing is a fundamental and essential functionality of a BiPCR. In some sense, a BiPCR can be regarded as a mobile manipulator, whose base may be changed and fixed in turn. During climbing, the robot fixes and supports itself with one of the two grippers served as the base, and moves the other one (the swinging gripper) to the target position, interchanging the roles of the two grippers in each climbing cycle. Hence to completely describe how a BiPCR climb in a spatial truss, we have to provide a series of discrete footholds and the continuous trajectories between adjacent footholds of the same swinging grippers. While the former define the gripping configurations of the BiPCR from the initial position to the destination, the latter determine the climbing motion of the robot in each climbing step. How to plan the footholds refers to climbing path planning or grasp pose planning of a BiPCR, which is out of the scope of this paper. Rather, given the footholds of the two grippers, how to plan the smooth and collision-free motion of the swinging gripper in one climbing step for a BiPCR in complex spatial trusses is an open and challenging issue and is the focus of this paper.

Climbing path planning of BiPCRs in spacial trusses has been investigated to some extent in the literature. The problem was converted into the classical traveling salesman problem considering the energy consuming during each climbing cycle in [6] and [7]. In [3], the trusses were discretized into a series of nodes and the sequence of clamping points from a given initial node to the destination one was planned by calculating the Dijkstra shortest distance and motion complexity as criteria. The above work on climbing path planning actually belongs to the category of foothold planning. However, to the best of our knowledge, single-step collision-free trajectory planning of a BiPCR climbing in complex spatial trusses has not been explored.

Climbing motion planning of a BiPCR in one climbing step is similar to that of an manipulator, since the robot is fixed and supported on a pole by the base gripper at a specific foothold, and the swinging gripper moves from its initial foothold (configuration) to the target one. Therefore, traditional algorithms for collision-free motion planning of manipulators, such as artificial potential field (APF) [8], probabilistic road map (PRM) [9], rapidly-exploring random tree (RRT) [10], and almost all the intelligent algorithms like genetic algorithm, particle swarm optimization (PSO) [11] can be utilized to generate the climbing trajectories. However, the motion planning of a BiPCR has its own features compared with that of an industrial robot. First, when the base of a BiPCR is changed and switched between the two grippers during climbing, those algorithms suitable for fixed base, such as PRM, will exhibit low efficiency. Second, some part(s) of the target pole is/are the graspable region(s) and other parts should be treated as obstacles, traditional algorithms like APF will encounter difficulties. Third, the role of a pole (target or obstacle) may interchange in different climbing cycles.

Considering the RRT algorithm has wide adaptation and good robustness to multiple degrees of freedom and dynamic environments, we address the problem of collision-free motion planning in one climbing step for BiPCRs in the spatial trusses, utilizing the Bi-RRT algorithm. The main contributions of this paper are as follows. On the one hand, the framework for climbing motion planning of BiPCRs with different DoFs is first built based on Bi-RRT. The proposed planning algorithm is adaptive to BiPCRs with different numbers of DoFs including five and six. For a 5-DoF BiPCR like Climbot-5D (hereafter we use Climbot-5D and Climbot-6D to represent the Climbot with five and six degrees of freedom, respectively) whose orientation is limited due to its special configuration, a simple but effective state expression method is presented to deal with the sampling, interpolation and metric processes, which also adapts to Climbot-6D. On the other hand, pre-process and post-process methods are proposed in this paper to guide the swinging gripper to move away from the starting foothold and to the target foothold. In addition, cubic B-spline curves are utilized to smooth the climbing trajectories.

## Theoretical analysis

### Description of a BiPCR in a truss

**Fig. 1:**
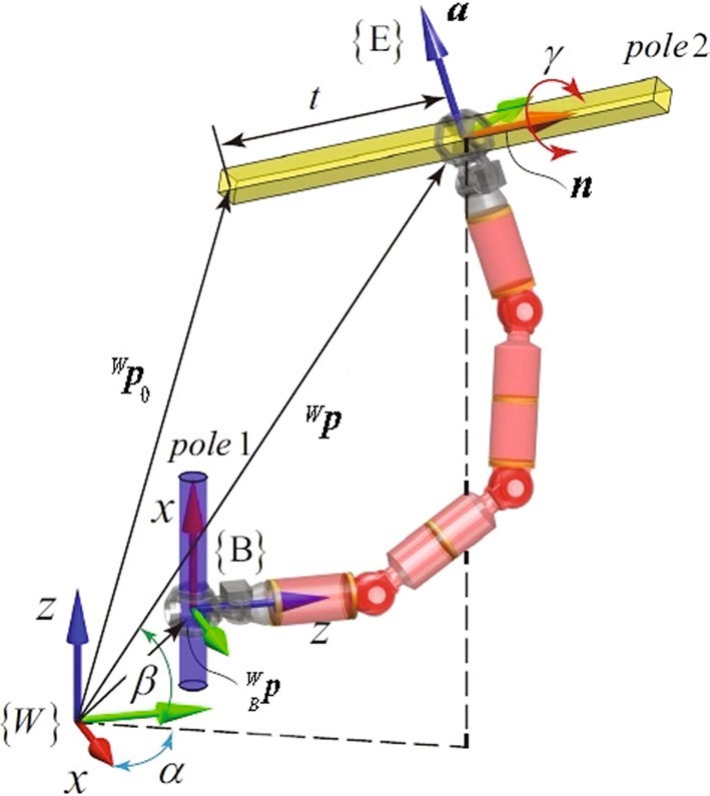
Description of the robot in trusses

In order to completely describe a BiPCR in a truss, we need to specify not only the position on a pole where the base gripper grasps, but also the configuration the robot achieves. Hence, two homogeneous transformation matrixes are needed—one is to locate the grasping base in the world frame (denoted as $_B^W{\varvec{T}}$), and another to indicate the swinging gripper (end-effector) with respect to the base frame of the robot (denoted as $_E^B{\varvec{T}}$), as shown in Fig. 1.

A conventional configuration description with homogeneous transformation matrix can be expressed as

1 $$\begin{aligned} {}_G^W{\varvec{T}} = \left[ {\begin{array}{cc} {{}_G^W{\varvec{R}}}&{}\quad {{}_G^W{\varvec{p}}}\\ 0&{}\quad 1 \end{array}} \right] \end{aligned}$$

where $\{ G\}$ represents the base frame attaching to the grasping gripper of the robot, ${}_G^W{\varvec{R}}$ and ${}_G^W{\varvec{p}}$ represent the rotation matrix and translation vector with respect to the world frame $\{ W\}$, respectively.

On the one hand, suppose that a pole is described in the world frame by the parametric equation as

2 $$\begin{aligned} {}^W{\varvec{p}} = {}^W{{\varvec{p}}_0} + {t}\cdot ^W{\varvec{d}} , \quad 0\le {t}\le {L} \end{aligned}$$

where ${}^W{\varvec{p}_0}$, ${}^W{\varvec{d}}$ and *L* are the reference point, the unit direction vector and the length of the pole, respectively. The gripping position on the pole must thus satisfy ${}_G^W{\varvec{p}} \in \{{}^W{\varvec{p}}\}$.

On the other hand, referring to Fig. 1, using notation $(\alpha , \beta , \gamma )$ of $Z{-}Y{-}X$ Euler angles, the orientation of a grasp can be calculated as

3 $$\begin{aligned} \left\{ {\begin{array}{l} {{}_G^W{\varvec{R}} = {{\varvec{R}}_Z}(\alpha ){{\varvec{R}}_Y}(\beta ){{\varvec{R}}_X}(\gamma )} \\ {\alpha = \arctan ({n_y}/{n_x})} \\ {\beta = - \arctan ({n_z}/\sqrt{1 - {n_z}^2} )} \end{array}} \right. \end{aligned}$$

where $\gamma$ denotes the grasping direction, which restrains the rotation around the pole, ${\varvec{n}}={[{n_x}\;\;{n_y}\;\;{n_z}]^T}$ is the unit direction vector of the pole to be grasped as shown in Fig. 1.

### Problem statement

In a single climbing step, collision-free motion planning involves three adjacent footholds, one of which determines the grasping configuration of the base gripper and the other two are the initial and the target configurations of the swinging gripper.^1^ A feasible and collision-free trajectory is to be found between the two footholds for the swinging gripper. The problem can be described as follows.

Suppose a BiPCR grasping on a pole with one of its grippers at ${}_B^W{{\varvec{T}}_i}$, the aim is to find the feasible trajectory for the swinging gripper moving from the initial foothold $^W_E{\varvec{T}}_{i-1}$ to the target one $^W_E{\varvec{T}}_{i+1}$. There should not be any collision between the robot and the climbing environment (the truss). Let $\tau$: [0,1] denote the trajectory and ${\varvec{q}} \in {{\varvec{R}}^n}$ denote the joint angles ($q_0$...$q_n$) of the robot, the single-step collision-free trajectory planning of the BiPCR can be modeled as

4 $$\begin{aligned} \left\{ {\begin{array}{l} {{\varvec{q}}_{\text {init}}} = IK \left( {}_B^W{{\varvec{T}}_i}^{ - 1}{}_E^W{{\varvec{T}}_{i - 1}}\right) \\ {{\varvec{q}}_{\text {goal}}} = IK \left( {}_B^W{{\varvec{T}}_i}^{ - 1}{}_E^W{{\varvec{T}}_{i + 1}}\right) \\ \tau (0) = {{\varvec{q}}_{\text {init}\;}} \\ \tau (1) = {{\varvec{q}}_{\text {goal}\;}} \\ {\tau :[0,1] \rightarrow {C_{\text {free}}}} \end{array}} \right. \end{aligned}$$

where $C_{\text {free}}$ refers to the collision-free configuration space and $IK(\,)$ represents the inverse kinematics.

### Pre- and post-process for easy trajectory planning

Since the grippers of a BiPCR are usually designed to grasp objects using two fingers with V-shaped grooves, the initial and target configurations of the swinging gripper in one climbing step are constrained with respect to the poles. As a result, the directions of the swinging gripper at the beginning and end of the climbing motion are restricted to be perpendicular to the corresponding pole. To improve the efficiency of the sampling-based algorithm, we propose a pre-process and a post-process to guide the swinging gripper to leave from the initial grasping configuration and approach the target configuration. To this end, a translation matrix is defined with respect to the gripper frame $\{ E\}$ as

5 $$\begin{aligned} {}_{P'}^E{\varvec{T}} = \left[ {\begin{array}{cccc} 1&{}\quad 0&{}\quad 0&{}\quad {\Delta x}\\ 0&{}\quad 1&{}\quad 0&{}\quad 0\\ 0&{}\quad 0&{}\quad 1&{}\quad {\Delta z}\\ 0&{}\quad 0&{}\quad 0&{}\quad 1 \end{array}} \right] \end{aligned}$$

where ${\Delta z}$ stands for the offset along the *Z* axis of $\{ E\}$ and ${\Delta x}$ for the translation along the pole (*X* axis of $\{ E\}$). ${}_{P'}^E{\varvec{T}}$ thus defines a new configuration of the swinging gripper with costant orientation.

With the translation matrix ${}_{P'}^E{\varvec{T}}$, we get a new homogeneous transformation in the pre-process describing the swinging gripper of the robot with respect to base frame $\{ B\}$ as

6 $$\begin{aligned} {}_{P'}^B{\varvec{T}} = {}_E^B{\varvec{T}}{}_{P'}^E{\varvec{T}} \end{aligned}$$

And the corresponding joint angles can be found as

7 $$\begin{aligned} {\varvec{q}}{'_{\text {init}}} = IK\left( _{P'}^B{\varvec{T}}\right) \end{aligned}$$

**Fig. 2:**
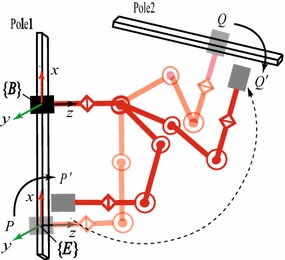
Pre- and post-process for planning

In the similar manner, a new goal configuration ${\varvec{q}}{'_{\text {goal}}}$ can be obtained. To ensure no collision occurs during the translation, collision detection should be conducted. After the pre-process, the original trajectory planning from *P* to *Q* is transformed to that from $P'$ to $Q'$, as shown in Fig. 2. In the post-process, the translational motion from *P* to $P'$ and from $Q'$ to *Q* is added to the planning output in turn to form a complete trajectory from *P* to *Q*.

The pre- and post-processes bring several benefits to the motion planning of a BiPCR including (1) expediting the convergence of the searching procedure with sampling-based algorithms, (2) simplifying collision check, no need to distinguish the grasped poles and the obstacle poles and (3) easy integration of collision-free trajectory planning and autonomous alignment of the gripper.

### Utilization of the reachable workspace

**Fig. 3:**
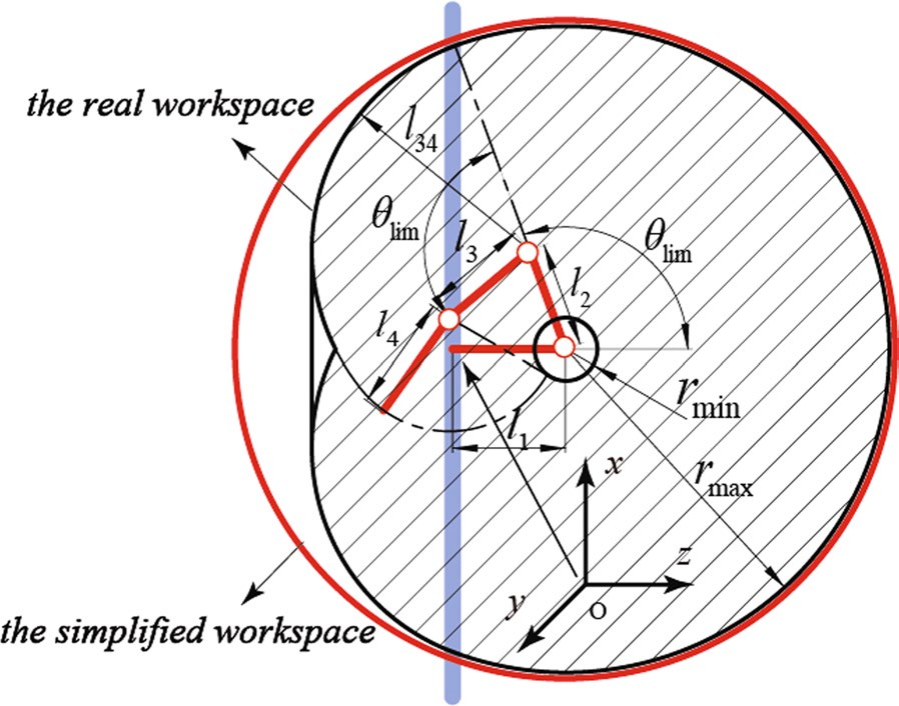
Profile of the reachable workspace

In this paper, the reachable workspace (as shown in Fig. 3) of a BiPCR is considered to simplify the planning problem. It is clear that only those poles inside the reachable workspace, rather than the whole truss, should be considered as the target or obstacle poles in the planning. Therefore, the reachable workspace contributes to filter the poles in order to accelerate the collision detection. Moreover, it is also utilized to define the sampling area.

Without loss of generality, taking the Climbot-5D for example, its reachable workspace can be described in polar coordinates with respect to the base frame $\{ \hbox {B}\}$, as shown in Fig. 3,

8 $$\begin{aligned} \left\{ {\begin{array}{l} {x = \rho \sin \theta \cos \varphi }\\ {y = \rho \sin \theta \sin \varphi }\\ {z = {Z_0} + \rho \cos \theta } \end{array}} \right. \end{aligned}$$

where $\theta$ and $\varphi$ are two parameters, ${Z_0}$ represents the offset along the *Z* axis of frame $\{ \hbox {B}\}$, and $\rho$ represents the radius of the workspace depending on the angle $\theta$,

9 $$\begin{aligned} \left\{ \begin{array}{ll} \rho = {l_{234}} = {\sum }_{i=2}^4{l_i}, &{} \theta \in [0,{\theta _{\lim }}] \\ \rho = \sqrt{{\rho _x}^2 +{\rho _y}^2 + {\rho _z}^2}, &{} \theta \in ({\theta _{\lim }},\pi ] \end{array} \right. \end{aligned}$$

where ${{l_i}}$ represents the length of the *i*-th link of Climbot-5D, $\theta _{\lim }$ represents the rotation angle limit of the T-type joint modules, ${\rho _x}$, ${\rho _y}$ and ${\rho _z}$ are obtained as

10 $$\begin{aligned} \left\{ {\begin{array}{l} {{\rho _x} = \left[ {l_2}\sin {\theta _{\lim }} + {l_{34}}\cos (\alpha + \theta - {\pi / 2})\right] \cos \varphi }\\ {{\rho _y} = \left[ {l_2}\sin {\theta _{\lim }} + {l_{34}}\cos (\alpha + \theta - {\pi / 2})\right] \sin \varphi }\\ {{\rho _z} = - {l_2}\cos {\theta _{\lim }} + {l_{34}}\sin (\alpha + \theta - {\pi / 2})} \end{array}} \right. \end{aligned}$$

where $l_{34} = l_3+l_4$ and $\alpha$ is an intermediate variable defined as

11 $$\begin{aligned} \alpha = \arcsin \frac{{{l_2}\sin (\theta - {\theta _{\lim }})}}{{{l_{34}}}} \end{aligned}$$

The complicated mathematic expression of the robot’s reachable workspace is inconvenient for application. Hence, the workspace is simplified here as a sphere with radius $\rho = l_{234}$ and center at $(0, 0, l_1)$ in the base frame $\{ \hbox {B} \}$, as shown in Fig. 3.

### Collision detection

Since the truss poles and Climbot links are cylindrical, collision detection is easily carried out through the calculation of the minimum distance between two line segments (the axes of a pole and a robotic link), which is divided into three steps as follows.

Step 1: describing the poles and the BiPCR in the world frame $\{ W\}$. Collision check can be conducted only under the condition that the robot and the obstacles are expressed in the same coordination frame. Without loss of generality, supposing “A” is an arbitrary point of the robot, its position can be calculated by the forward kinematics with respect to frame $\{ B\}$ as ${^B{\varvec{p}_A}}$, then transformed to $\{ W\}$ by $^W{\varvec{p}_A} = {}_B^W{\varvec{T}}^B{\varvec{p}_A}$.
Step 2: finding the pole segments within the simplified reachable workspace of the robot. The algorithm to calculate the line segment inside a sphere can be found in [12]. This intersecting segment can be described by two points with parameters ${{t_1}}$ and ${{t_2}}$ respectively, having the form as
  12 $$\begin{aligned} {{{\varvec{p}}_i}} = {{{\varvec{p}}_0}} + {t_i} {\varvec{d}} \end{aligned}$$
  where ${{{\varvec{p}}_0}}$ and ${\varvec{d}}$ represent the reference point and the unit direction vector of a line segment.
Step 3: computing the minimum distances between the remaining poles and links of the robot, and comparing with the threshold (the sum of radii of the pole and the robotic link). Collision is reported when the computed distance is less than the threshold; otherwise, there is no collision between the poles and the robot. The pseudo-codes of the algorithm are listed in Algorithm 1. In the algorithm, Seg2SegDist means the function calculating the Euclidean distance between two spatial line segments.

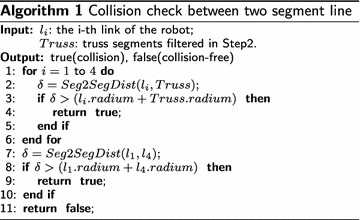


## The motion planning algorithm

### Bi-RRT algorithm 

**Fig. 4:**
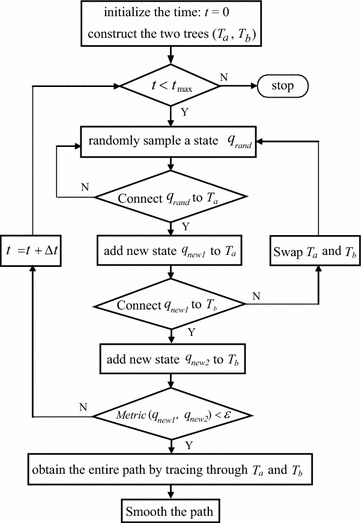
Flowchart of the algorithm

The RRT algorithm was first proposed by Lavalle [13] in 1998 and has been widely applied in the field of robotics since then. RRT-based algorithms may be classified into two categories: single directional and bidirectional RRTs (single-RRT and Bi-RRT). Considering the higher searching efficiency, we adopt the Bi-RRT algorithm in this paper, as shown in Fig. 4, with the pseudo-codes listed in Algorithm 2. 
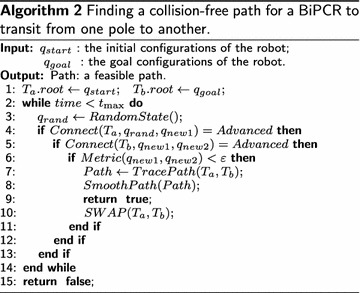

### Constraints on grasping orientation

It is well known that a manipulator with six DoFs may reach arbitrary configuration in its workspace. The configuration of a 6-DoF BiPCR can be described by a 3-D position and a 4-D unit quaternion, similar to that of an industrial robot.

Unfortunately, a 5-DoF BiPCR is unable to reach arbitrary orientation. As a result, if the Euler angles or quaternions are utilized to describe its orientation and to interpolate, the reachability of a desired configuration cannot be guaranteed. In other words, the computed configuration of the robot may not be accurate when the inverse kinematics presented in [4] is used directly.

**Fig. 5:**
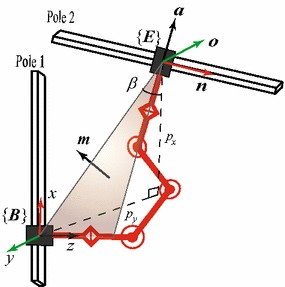
Geometric relationship between the robot and the poles

Due to the special kinematic structure of Climbot-5D, the robot’s links are always restricted in a plane (referred as “robot plane,” the shaded triangle area in Fig. 5). As a consequence, the following constraints on orientation must be satisfied

13 $$\begin{aligned} \left\{ {\begin{array}{l} {{\varvec{a}} \cdot {\varvec{n}} = 0} \\ {{\varvec{a}} \cdot {\varvec{m}} = 0} \\ {||{\varvec{a}}|| = 1} \\ {\tan \beta = {p_y}/{p_x}} \end{array}} \right. \end{aligned}$$

where ${\varvec{m}}={[- \sin \beta \;\;\cos \beta \;\;0]^T}$ represents the normal vector of the robot plane and ${p_x}$, ${p_y}$ represent the position projection components with respect to the base frame $\{B\}$. Therefore, the grasping orientation ${}_E^B{\varvec{R}}$ can be calculated through Eq. (13) with the known grasping position. In other words, once the grasping position and the direction of a pole (the vector ${\varvec{n}}$) are given, the orientation of Climbot’s swinging gripper is determined uniquely.

As a result, we may specify the configuration of a 5-DoF BiPCR using a position vector and a direction vector (six dimensions in total) and describe that of a 6-DoF BiPCR using a position vector and a unit quaternion (seven dimensions in total).

### Random sampling

In order to guarantee the uniform distribution of the sampling, and to take into account the multiple gaits of a BiPCR, we sample the configuration of a BiPCR in the workspace with respect to the base frame.

On the one hand, recalling Fig. 3, the position is sampled in the robot’s reachable workspace as

14 $$\begin{aligned} {\varvec{p}} = {\varvec{p}}_{\min } + r({\varvec{p}}_{\max }-{\varvec{p}}_{\min }), \end{aligned}$$

where ${\varvec{p}}\in {\varvec{R}}^3$, $r \in [0, 1]$, ${\varvec{p}}_{\max }=l_{234}{[1\;\;1\;\;1]^T}$ and ${\varvec{p}}_{\min } = -l_{234}{[1\;\;1\;\;1]^T}$, ensuring that

15 $$\begin{aligned} {r_{\min }} < \parallel {\varvec{p}}\parallel < {r_{\max }}, \end{aligned}$$

where ${r_{\min }}$ and ${r_{\max }}$ represent the radii of the inner inaccessible sphere and the outer reachable sphere, respectively. Considering the center of the reachable workspace has an offset to the origin of the base frame, the sampled position should be finally moved by

16 $$\begin{aligned} {\varvec{p}}^\prime = {\varvec{p}} + [0 \,\, 0 \,\, l_1]^T. \end{aligned}$$

On the other hand, since we use vectors with different dimensions to describe the orientation of the swinging grippers of 5-DoF and 6-DoF BiPCRs, two methods are utilized to sample the orientation component, respectively. For a 6-DoF BiPCR, a simple sampling algorithm in SO(3) performs well in sampling unit quaternion [14]. For a 5-DoF BiPCR, we need to sample the direction of a virtual pole (the ${\varvec{n}}$ component) and then calculate the grasping orientation by Eq. (13). To this end, the HEALPix algorithm [15] is employed to generate two angular parameters ($\theta , \varphi$) in spherical coordinates, which is then transformed to a 3-D directional vector by ${\varvec{n}}={[\cos \theta \;\;\sin \theta \cos \varphi \;\;\sin \theta \sin \varphi ]^T}$.

So far, through sampling we have achieved a 6-D random state (a 3-D position and a 3-D direction vector) for a 5-DoF BiPCR and a 7-D random state (a 3-D position and a 4-D unit quaternion) for a 6-DoF BiPCR, respectively.

### Distance metric

The distance metric is very important for sampling-based algorithms. The most simple and commonly used metric can be defined as

17 $$\begin{aligned} \rho ({{\varvec{q}}_0},{{\varvec{q}}_1}) = {\omega _p}||{{\varvec{p}}_0} - {{\varvec{p}}_1}|| + {\omega _r}f({{\varvec{R}}_0},{{\varvec{R}}_1}), \end{aligned}$$

where ${\varvec{p}}$ and ${\varvec{R}}$ indicate the position and the rotation components of the configuration, respectively, ${\omega _{\text{ p }}}$ and ${\omega _{\text{ r }}}$ represent their weighting scales, $|| \, ||$ means the standard Euclidean norm in 3-D and *f*() stands for the measurement between two orientation matrices.

Considering that the inner product of two quaternions or vectors indicates the difference between them, one option to define the function *f*() is

18 $$\begin{aligned} \psi = \arccos ({\text{ dot }}({\varvec{R}}_0,{\varvec{R}}_1)), \end{aligned}$$

where ${\varvec{R}}_0$ and ${\varvec{R}}_1$ are quaternions or 3-D vectors.

Since the value of the rotation “distance” is limited to be less than $\pi$, we can specify the rotation distance weight as ${\omega _r} = 1/\pi$ to normalize the orientation distance. Correspondingly, the weight for position distance can be set as ${\omega _p} = 1/(2l_{234})$. Hence, the configuration distance is limited in [0, 1] by

$$\begin{aligned} \rho ({{\varvec{q}}_0},{{\varvec{q}}_1}) = \frac{{{\omega _p}}}{2}\left\| {{{\varvec{p}}_0} - {{\varvec{p}}_1}} \right\| + \frac{{{\omega _r}}}{2}\arccos({\text{ dot }}({{\varvec{R}}_0},{{\varvec{R}}_1})). \end{aligned}$$

### Configuration interpolation

When interpolating between two configurations, it is usually separated into two parts corresponding to the position and orientation components. For the position component, a simple linear interpolation is suitable. As for the orientation component, it depends on the inner product of the two unit quaternions or 3-D vectors. If the orientations are close enough (their inner product is bigger than the pre-defined threshold), the linear interpolation algorithm is applied. Otherwise, the spherical linear interpolation algorithm is carried out, which is able to ensure the smooth interpolation between two configurations along geodesics.

### Motion smoothing

Sampling-based planning may sometimes generate jerky and unnatural trajectories whose first derivatives are not continuous [16], which results in non-smooth motion or vibration of the robot. Therefore, motion smoothing is necessary. We utilize cubic B-spline fitting in this paper considering its sufficient flexibility and high-order smoothness.

**Fig. 6:**
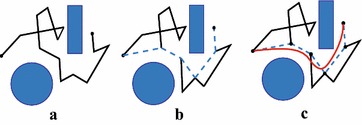
Overview of the smoothing scheme: **a** the original jerky piecewise linear path; **b** path after the linear shortcut process; **c** path after fitting with the cubic B-spline

The smoothing algorithm is illustrated in Fig. 6. A linear shortcut of the original piecewise linear path is first carried out to obtain a shorter path [the dash line in (b)]. The vertices of the dash line are then taken as the control points, and a non-uniform cubic B-spline is constructed as the final path [the red solid curve in (c)].

Note that we set double coincidence points at the two ends of the piecewise linear path to ensure the cubic B-spline curve passing them exactly. We set also weights for each control point to adjust the shape of the cubic B-spline curve. The larger the weight is, the closer the curve gets to the control point. As discussed above, the path is composed of a series of configurations of the robot. While the position portions of the configurations are fitted with a cubic B-spline in 3-D space, the orientation portions are calculated by interpolating between every two adjacent configurations at the vertices of the shortcut path. In configuration check along of the path, the position portion may be changed by adjusting the weights of the control points of the cubic B-spline to satisfy the inverse kinematics and ensure collision avoidance.

## Simulations and results

To verify the effectiveness of the theoretical analysis and the presented algorithms above, simulations are conducted in this section. The trusses are composed of cylindrical and squared poles with a diameter of 60 mm in arbitrary orientation in 3-D space. Both Climbot-5D and Climbot-6D are employed to test the proposed algorithm.

**Fig. 7:**
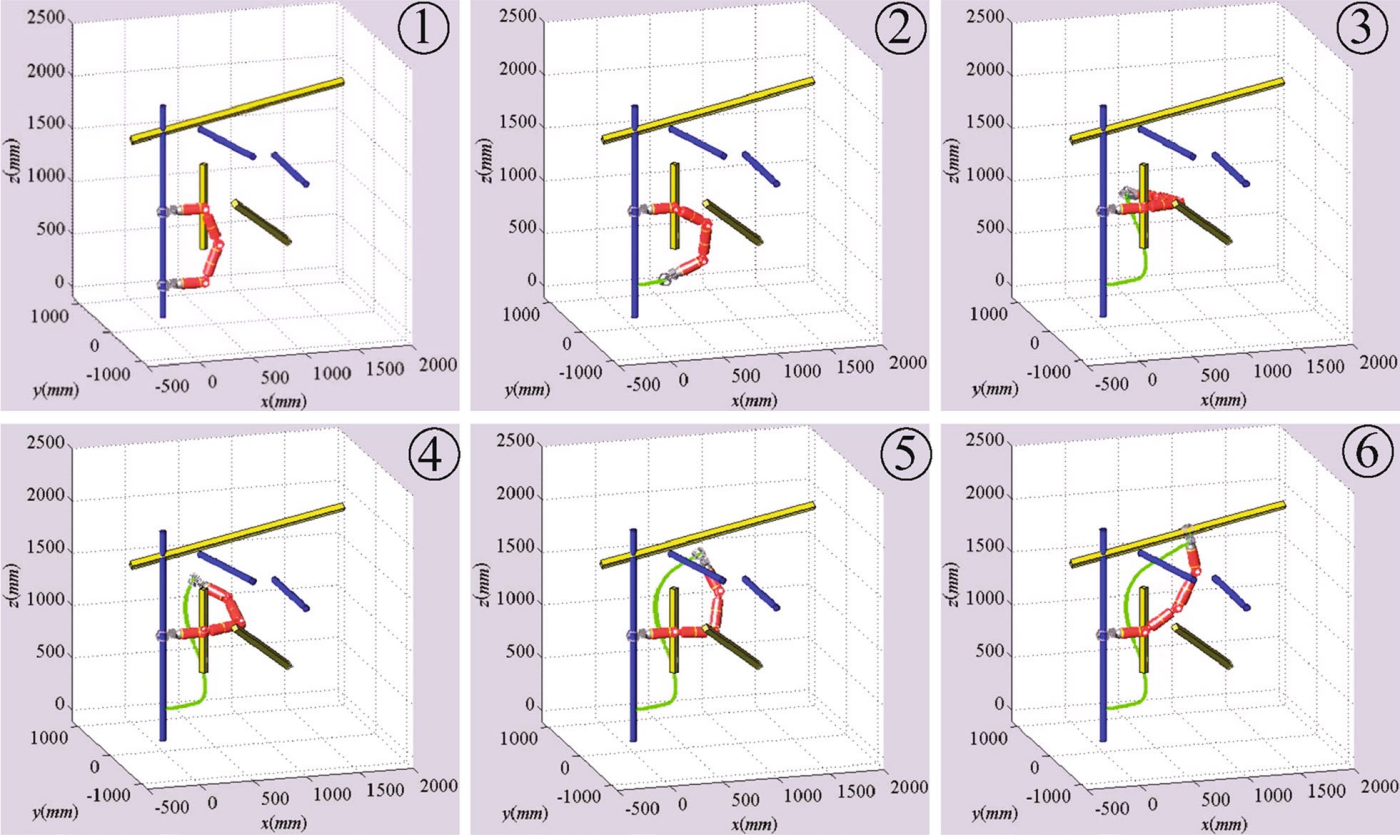
Simulation result with Climbot-5D

**Fig. 8:**
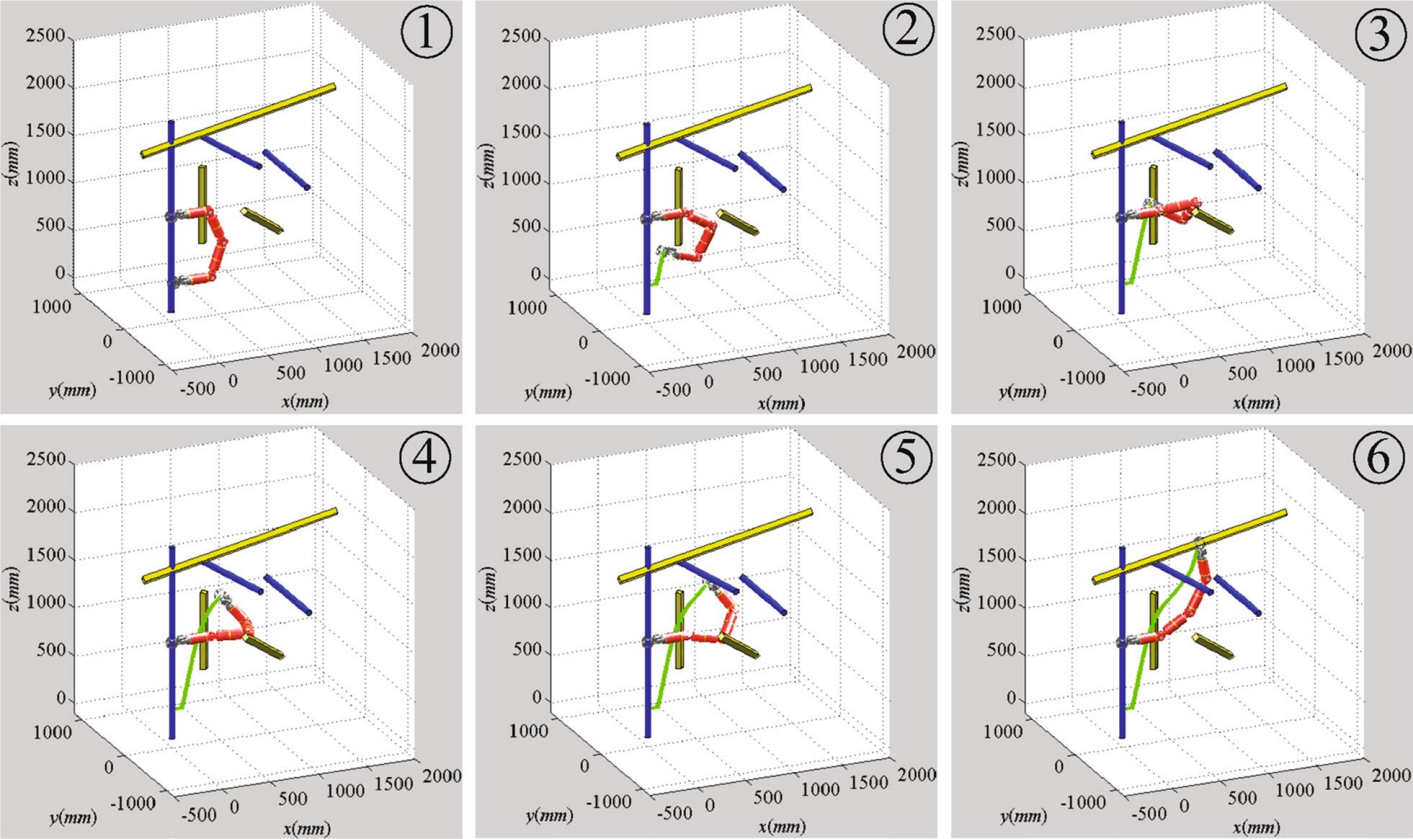
Simulation result with Climbot-6D

The step length for state verification in the algorithm is set to 40 mm, less than the diameters of poles, to make sure that the robot will not across a pole. The maximum node number of the two RRT trees is set to 500, and the goal-bias sampling probability is set to 0.05. Figures 7 and 8 show the simulation results with Climbot-5D and Climbot-6D, respectively.

 The simulations are conducted 50 times in the same truss. A comparison between the simulations with the two BiPCRs is shown in Table 1. It can be seen from the simulations that the Bi-RRT algorithm has excellent performance on the motion planning of the BiPCRs. The result with Climbot-5D also demonstrates the effectiveness of the processing method for sampling, distance calculation and interpolation. In addition, the simulation with Climbot-6D consumes less time than that with Climbot-5D, and has a shorter path length, owing to better dexterity with more degrees of freedom.

**Table 1:** A comparison between the simulations

BiPCR	Time (s)	Iteration times	Tree nodes	Path length (m)
Climbot-5D	4.46	130	104	2.93
Climbot-6D	0.593	32	38	1.21

## Conclusions and future work

Autonomous climbing is an essential function to carry out high-rise tasks with BiPCRs. Collision-free motion planning of BiPCRs in spatial trusses is an open problem, which has been addressed in this paper as a fundamental step to autonomous planning of climbing motion. A sampling-based algorithm, Bi-RRT, has been ultilized for single-step collision-free trajectory planning for BiPCRs. With appropriate description of a BiPCR in a truss, climbing motion planning has been conducted in a manner similar to that of a manipulator. The constraint on grasping orientation and the basic operations such as sampling, configuration distance calculation and interpolation have been discussed to facilitate the application of RRT. To expedite the convergence of the Bi-RRT algorithm, pre-process and post-process have been presented to deal with leaving from the starting point (the initial grasp configuration) and approaching the goal point (the final grasping configuration) of the swinging gripper. A method to smooth the piecewise linear jerky trajectory generated by the Bi-RRT algorithm has been proposed by utilizing cubic B-spline curve fitting. Simulations have verified the effectiveness of the theoretical analysis and the presented algorithm. The algorithm is general and universal for motion planning of other robots including manipulators and biped wall-climbing robots.

In the future, the algorithm will be integrated into the robot’s multi-layered planner for online climbing path and motion planning. And the dynamic constraints like the limit of joint velocity, acceleration and torque will be taken into account.

## Footnotes

1 The foothold of the base gripper may be at the end or in the middle of the three footholds, depending on the climbing gait—if the inchworm gait is used in the climbing step, the foothold of the base gripper is at the end, since the (front and rear) order of the two grippers are not changed; otherwise, if the turning-around gait or the flipping-over gait is employed, it is in the middle of the three footholds, since the order of the two grippers interchange [4].
